# Concerning the Treatment of Bubo by Extirpation of the Glands

**Published:** 1887-12

**Authors:** W. H. Heath

**Affiliations:** Surgeon to the Sisters of Charity Hospital, Buffalo, N. Y.


### *CONCERNING THE TREATMENT OF BUBO BY EXTIRPATION OF THE GLANDS.*

Referring also to a Case of Gangrene of the Scrotum following an Operation for the Radical Cure of Hernia.

By W. H. HEATH, M. D.,
Surgeon to the Sisters of Charity Hospital, Buffalo, N. Y.

The treatment of bubo depends upon whether the condition be acute or chronic. To the latter condition, which may be a sequel of an acute process, or indolent and slow from its incipiency, reference alone is had.

Irrespective of the character, duration or intensity of the venereal irritation, or of the influence of constitutional condition, or whether the inflammatory process be primarily glandular or peri-glandular, chronic buboes usually present one of the following conditions, and may be so classified:

1st. As unhealthy, irregular ulcers, with undermined or everted edges, and embedded in which are found one or more necrotic glands, the whole a consequence of suppuration attended with considerable destruction of tissue.

2d. As chronic inflammatory enlargements, composed of a number of glands in different stages of hyperplasia and breaking-down, with considerable peri-glandular induration and suppuration, the whole mass and overlying tissue being matted together, discharging a variable amount of watery-like or badly-formed pus through one or more fistulous openings.

3d. The same condition at an earlier stage, and which may be described as indolent glandular enlargements, only differing from the former in that there is no perceptible breaking-down or suppuration, and little or no involvement of surrounding tissue. (Two facts worthy of note, in connection with both these conditions, may be stated as evidence of the strong influence of heavy labor and strains, viz., the not infrequent implication of one or more glands below Poupart's ligament, and the occasional absence of anything like recent venereal disease.)

4th. As old, indurated, granular, sinuous tracts, found usually as a sequel of the above.

5th. As serpiginous bubo, rather rare in my experience.

Lastly, the glandular induration accompanying the initial lesion and secondary stage of syphilis.

These different conditions, with possibly few exceptions, are best treated by *removal of the diseased glands*, a procedure of no magnitude or great danger. It possesses the advantage not only of shortening the treatment, but preventing a repetition of the trouble, which frequently happens, as it is a clinical fact that glands, once inflamed, do so again on slight provocation, and suppurate with great rapidity.

Irregular, unhealthy ulcers, containing remnants of glands, should be thoroughly scraped with dull curette, or handle of a cartilage knife, and all detritus removed, undermined and everted edges clipped away with scissors, and granulation from the bottom induced on a fresh and healthy basis.

Those inflammatory enlargements, composed of breaking-down, hyperplastic glands and peri-glandular connective tissue, require radical treatment. The sinuses should be laid open, and every affected gland removed, shreds and broken-down tissue scraped or clipped away, every burrowing pocket opened out, after which the cavity should be thoroughly cleansed, and dressed with a stimulating and antiseptic dressing.

When they come under treatment at an earlier stage, as a mass of hyperplastic glands, without involvement of the adjacent tissues and no evident suppuration, in the majority of instances the same line of treatment will be found the most satisfactory. In these cases, the general practice of using compression, absorbents, counter-irritants, injections of carbolic acid, iodide of potash, and other substances (a painful and disappointing method), while successful and efficacious sometimes, as often fails, suppuration setting in as a result of, or in spite of the treatment, rendering extirpation even then the best method.

Examination of these glands when removed under the conditions stated, show some in a state of induration, others enlarged by newly-formed tissue, and containing one or more points of softening, evidence of a series of interrupted irritations extending over some period of time, altogether a condition more apt to go through a tedious process of disintegration and slow discharge, than absorption under any mode of treatment.

Old sinuses should be handled as in other situations, laid freely open, their surfaces stimulated or changed by scraping or caustic, and packed to heal from the bottom. In these cases, it is sometimes necessary to dissect out a puckered mass of hard cicatricial tissue. Good results may occasionally be obtained by dilating with tents, scraping and packing with glycerine.

Slow serpiginous ulceration is often remedied by completely dissecting out or scraping the ulcer, and starting the reparative process afresh, constitutional treatment always being persisted in.

True syphilitic bubo, by which is understood the adenopathy co-existing with the initial lesion and second stage of syphilis, requires no local treatment, except occasionally in scrofulous subjects, when it is determined by the peculiarity of the case.

In the removal of diseased inguinal glands, it may be stated that the incision should be free, and when practicable made in a line of the axis of the limb, thus obtaining better drainage, less disturbance to the wound from movement, less injury to the arteries or their small branches, and finally, better cicatrix. After exposure of the glands, their enucleation will be more judiciously accomplished with the fingers or knife-handle, rather than with the blade; in fact after the first incision, what very little cutting is necessary is best done with scissors.

A procedure of this kind among tissues already the seat of inflammatory changes, need not be conducted with the idea of obtaining other healing than by granulation from the bottom. By thorough cleansing however, the free use of antiseptics and antiseptic dressings, the subsequent course is materially influenced. Undue inflammation and irritation is prevented, suppuration very much limited, healing expedited, and systemic infection diminished if not prevented, a rise of temperature being rare. To be more particular at the risk of being elementary, I would say that, after thoroughly cleansing the wound, a very good way is to make an application of chloride of zinc, ten to twenty grains to ounce, dust iodoform, more particularly in pockets or recesses, pack the cavity lightly with a fold of antiseptic gauze, leaving the end between the lips of the wound, and over all a dressing of sublimated wood-wool, conveniently held in a bag of antiseptic cheese-cloth; and lastly, a firm spica.

The use of wood-wool in general surgery needs no mention here, but no dressing appears better adapted to the purpose and condition in question. Under its use, the wound need be disturbed but little, change of dressing being necessary but once in three or four days, granulations thrive, drainage by absorption is complete, and the parts are kept in a pure and healthy state.

The dangers now and then alluded to, and cited in objection to this treatment, appear more imaginary than real.

Hemorrhage from the superficial circumflex illi, external epigastric or external pudic, when cut, is readily controlled by tortion or ligature, oozing from overvascular tissue, checked by hot water or pressure of the dressing. Secondary bleeding is not more liable here than elsewhere, has never occurred in my cases, would not be serious, and could be easily controlled.

Important adjacent structures are protected by the deep facia, and particularly by the operator's anatomical knowledge and care, without which any operation may be dangerous; in fact, any injury to them would seem inexcusable. Pus could not enter the peritoneal cavity via the external abdominal ring and inguinal canal, possibility of which has also been suggested, unless the patient had a hernia, as no canal exists save as an abnormal condition, and besides, no necessity exists for exposing such aperture.

Gangrene of the scrotum following the operation in three cases, as reported by Drs. Winslow and Jones, (*Annals of Surgery*, January, 1886,) is I should say, an almost unprecedented or unobserved occurrence, though so formidable as to be borne in mind and its cause carefully studied.

In the absence of any other explanation, its most likely cause appears trophic, the result of injury to the ilio-inguinal nerve, rather than to infiltration from gravity, extension of inflammation or interference with the blood supply. As a contribution to the study of the subject, and the possibility of its following operative treatment for inguinal hernia, the possibility of which has been suggested, though no cases appear to be reported, it seems appropriate to place on record here the occurrence of this unlooked for complication after an operation for the radical cure of an irreducible hernia which happened in my practice a few years ago.

The patient, a man forty-five years of age in perfect health, apparently, had suffered for many years from an irreducible inguinal hernia, mainly omental in character. Its large size and steady increase interfered very much with his business, causing considerable mental anxiety and also abdominal disturbance, in consequence of which he was referred to me for treatment.

The operation presented no unusual features, and was conducted with strict antisepsis. It consisted in exposing the parts, ligating and removing the altered omentum and thickened sac, and closing the external ring by sutures, which included the pedicle. Thirty-six hours later, œdema and swelling of the scrotum set in, followed by gangrene and death.

Examination afterwards showed the gangrene to be independent of the wound, which was sweet, healthy, and almost entirely closed by primary union.

The explanation of this unhappy accident was extremely difficult at the time, and could only be accounted for under the supposition of injury to the ilio-inguinal nerve, or a condition of the kidney, not revealed by examination of the urine, or, as a remote possibility, both.

A complete autopsy not being allowed, these organs could not be examined, though there was no reason for believing them other than normal.

In conclusion it remains to be stated that the advantages of this method of treating chronic bubo, as found by the writer in an experience of over one hundred cases, is that it is radical and thorough, the duration of treatment shortened and recurrence precluded.

